# All‐Optical and Label‐Free Stimulation of Action Potentials in Neurons and Cardiomyocytes by Plasmonic Porous Metamaterials

**DOI:** 10.1002/advs.202100627

**Published:** 2021-09-05

**Authors:** Giulia Bruno, Giovanni Melle, Andrea Barbaglia, Giuseppina Iachetta, Rustamzhon Melikov, Michela Perrone, Michele Dipalo, Francesco De Angelis

**Affiliations:** ^1^ Plasmon Nanotechnologies Istituto Italiano di Tecnologia Genova 16163 Italy

**Keywords:** cardiomyocytes, complementary metal‐oxide semiconductor multielectrode arrays, metamaterials, microelectrode arrays, neurons, plasmonic optical stimulation, plasmonics

## Abstract

Optical stimulation technologies are gaining great consideration in cardiology, neuroscience studies, and drug discovery pathways by providing control over cell activity with high spatio‐temporal resolution. However, this high precision requires manipulation of biological processes at genetic level concealing its development from broad scale application. Therefore, translating these technologies into tools for medical or pharmacological applications remains a challenge. Here, an all‐optical nongenetic method for the modulation of electrogenic cells is introduced. It is demonstrated that plasmonic metamaterials can be used to elicit action potentials by converting near infrared laser pulses into stimulatory currents. The suggested approach allows for the stimulation of cardiomyocytes and neurons directly on commercial complementary metal‐oxide semiconductor microelectrode arrays coupled with ultrafast pulsed laser, providing both stimulation and network‐level recordings on the same device.

## Graphical abstract

The complementary metal‐oxide semiconductor (CMOS) multi‐electrode array coupled with picosecond near infrared laser pulses is capable of stimulating electrogenic cells. When irradiated, the plasmonic material covering the CMOS microelectrode arrays (MEAs) electrodes produces nanoampere currents due to the hot electron injection in the cleft. This can specifically elicit cardiomyocytes and neuronal cells directly in situ without genetic or thermal tools.

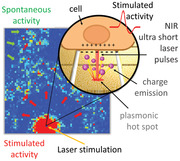

## 1 Introduction

Cellular activity stimulation is a pivotal technique for restoring the physiological heart contraction, for exploring the electrical functionality of cardiac and neuronal cells in vitro, for investigating the effects of molecular entities on the cells, and for the treatment of patient's cardiovascular or neurological debilitating conditions.^[^ ^1^, ^2^ ^]^ Electrical^[^ ^1^, ^2^ ^]^ and optogenetic^[^ ^3^, ^4^, ^5^, ^6^, ^7^, ^8^ ^]^ methods are the gold standard approaches for cell electrophysiological stimulation. However, they still present severe limitations in terms of invasiveness and compatibility with pharmacological screenings.^[^ ^9^ ^]^

As a valid alternative, nongenetic photostimulation does not require alteration of the cellular structure while still preserving high spatial resolution. In this context, light‐responsive materials have been explored as substitutes of the traditional genetic‐based optical stimulation techniques. Silicon nanowires,^[^ ^10^ ^]^ gold nanorods,^[^ ^11^ ^]^ semiconducting polymers,^[^ ^12^, ^13^, ^14^ ^]^ nanoparticles,^[^ ^15^ ^]^ and quantum dots^[^ ^16^ ^]^ represent the cutting‐edge structures for neuro‐ and cardiac‐modulation in fundamental biological studies and prosthetic applications. For instance, photothermal effect due to the irradiation of gold nanoparticles via 532 nm picosecond laser pulses proves to induce calcium oscillations in cardiomyocytes that leads to change in contraction rate of these cell aggregates.^[^ ^15^ ^]^ This approach can reduce the laser power required to excite neurons and can increase the stimulation selectivity, thus decreasing the potential harmful effect on cell health and integrity of the pure thermal stimulation methods. As recently indicated in the work of DiFrancesco et al.,^[^ ^17^ ^]^ engineered light‐sensitive compounds (photo‐switches), when irradiated with visible light, demonstrate to induce a transient hyperpolarization followed by a delayed depolarization that causes firing activity in neurons loaded with these molecules. In parallel, Tian and co‐workers demonstrated optical neuromodulation^[^ ^18^ ^]^ and cardiac action potential (AP) eliciting^[^ ^19^ ^]^ with silicon‐based materials. In contrast to pure photothermal materials, these devices meet the requirements of being minimally invasive and better integrated with target biological systems.

Introducing cell activity stimulation in drug screening assays is also expected to improve the efficiency of the early stages of drug discovery, leading to broader and more accurate analyses of drug candidates. In a recent work, Zeng et al.^[^ ^20^ ^]^ highlighted the importance of introducing external pacing within the safety evaluation of drug candidates, in order to cover a wider frequency spectrum of human heart activity in the same experiment. In this regard, a platform aiming at offering an accurate pharmacological profiling requires the ability to modulate dynamically the frequency of the cellular activity together with the simultaneous monitoring of cellular responses. In this direction, patch clamp offers accurate readout of the entire range of currents produced by electrogenic cells at single‐cell level. In recent years, the automated patch clamp has allowed also for high‐throughput assays for cardiovascular channelopathies modeling and drug safety pharmacology.^[^ ^21^, ^22^ ^]^ Despite these improvements, patch clamp remains however limited to studies of isolated cells. The parallel electrophysiological screening and mapping of cellular activity in complex networks has been achieved over the years with microelectrode arrays (MEAs). This technique is one of the main methods used in basic biological studies and, thanks to recent improvements in nanofabrication technology, it is becoming promising also in pharmaceutical research for evaluating drug effects on target cells.^[^ ^23^, ^24^, ^25^ ^]^ In this framework, a recent study from Abbott et al. demonstrates the capability of their nanofabricated complementary metal‐oxide semiconductor (CMOS) neuroelectronic interface to perform simultaneous intracellular recording and stimulation of a whole rat neuronal network.^[^ ^26^ ^]^ This array of 4906 platinum‐black electrodes, through the application of faradaic current, could access the intracellular compartment allowing a patch‐clamp‐like recording and stimulation of the neurons. Besides the high accuracy, this device requires several sophisticated and time‐consuming fabrication steps, hindering the possible commercial scaling. In response to this necessity, the optical stimulation methods should aim at the easy and effective integration with MEA biosensors for increasing their potential exploitation in pharmaceutical research.

In recent works, we have shown that plasmonic nanostructures and porous metamaterials can efficiently emit “hot” electrons under light excitation with ultrafast pulsed lasers (femtosecond–picosecond regime)^[^ ^27^, ^28^ ^]^ in the near infrared (NIR) range of the spectrum. When cells are cultured on the plasmonic materials, the emitted electrons can strongly interact with the cellular membrane, producing useful effects in a variety of applications.^[^ ^28^, ^29^, ^30^, ^31^ ^]^ Notably, the use of NIR lasers (*λ* = 1–1.5 µm) enables deep tissue penetration even in dense media while preventing cell damage. In fact, the term “hot electron” does not refer to temperature increase or to heat accumulation that may damage the cells. It rather indicates that the emitted electrical charges are out of thermal equilibrium because the laser excitation pulse is much faster than the typical time constant of thermalization processes. This term also helps to discriminate these electrons from conventional photo‐electrons.

Among the mentioned applications, it was demonstrated the capability of porous electrodes to open nanopores in the cellular membrane and to record intracellular‐like action potentials, paving the way to a more reliable monitoring of electrophysiological cellular activity and evaluation of pharmacological and toxicological effects on commercial MEA devices.^[^ ^29^ ^]^

Here, we demonstrate that ultrafast pulsed laser excitation of porous metamaterials is also an efficient and noninvasive stimulation tool for eliciting action potentials in cardiomyocytes and primary neurons. The stimulation stems from the high capacitive and faradaic photocurrents generated by hot electrons at the metamaterial/cell interface under laser excitation. Being the porous metamaterials already integrated on MEAs, the cells can be concurrently stimulated and recorded within complex cultures on the same device. Since the laser is focused with a spot size below 2–3µm in diameter and hot electrons are generated only in this region, it provides very high spatial resolution and can excite single cells, from which the activity propagates to the rest of the culture and is recorded by the MEA device (panel 1A,B).

Thus, this approach has high potential for cell stimulation at single‐cell level on high‐density/high‐resolution recording biosensors, opening the way to new methodologies for studying cardiomyocytes and neurons under various stimulation protocols. Our results pave the way to the use of opto/plasmonic cell stimulation for drug screening assays. In fact, this technology can be integrated with commercial CMOS‐MEAs without any modification of the platform, rendering the method accessible for real‐world applications in the pharmaceutical and biomedical industries.

## 2 Results and Discussion

### 2.1 Plasmonic Metamaterials as Hot Charges Emitters for Cellular Stimulation

For cellular stimulation, we exploit the well‐established photo‐electrochemical mechanism based on the combination of capacitive–faradaic currents at the cell/material interface.^[^ ^10^, ^17^, ^18^, ^32^, ^33^ ^]^ In this approach, the local reduction of the extracellular potential and the local production of redox reactions are responsible for stimulating action potentials.^[^ ^18^ ^]^

**Figure 1:**
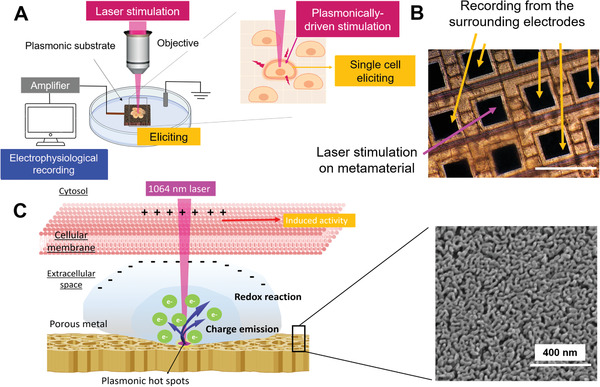
Optical stimulation of cells with plasmonic metamaterials. A) Principle of activity stimulation by single‐cell excitation with ultrafast pulsed laser. Inset: the concept of plasmonically driven stimulation. B) Bright field image of the HiPSC‐CMs cultured on a CMOS‐MEA device. The arrows show the typical configuration in which an electrode is used for optical stimulation and the surrounding electrodes are used for recording. Scale bar: 30 µm. C) Detail of the optical charge emission induced by metamaterials when irradiated by NIR laser. Inset: SEM image of the porous material. Scale bar: 400 nm.

Whereas stimulating photo‐generated currents are typically obtained by hole–electron pairs in silicon nanomaterials,^[^ ^18^ ^]^ the plasmonic capacitive–faradaic currents can be induced by ultrafast pulsed laser radiation of plasmonic nanostructures^[^ ^27^, ^34^ ^]^ and metamaterials.^[^ ^29^ ^]^ The typical configurations employ focused laser sources in the NIR region with pulses in the femto–picosecond range. In case of nanoporous metamaterials, the key factor enabling cell stimulation is that the energy collected from the incident light is efficiently confined into the nanopores of the metamaterial, generating plasmonic hot spots of intense electric field that results in charge photo‐emission at the interface (**Figure** **1**C). This intrinsic characteristic leads to an extremely localized process that does not affect the surrounding regions. The high energy of hot electrons produced in the first ≈10–100 femtoseconds provide strong reactivity and lead to very efficient redox chemistry. We remark that the term hot spot does not refer to heating, but to increased field intensity.

To support this hypothesis, we first characterize and quantify the capacitive–faradaic currents at the interface between nanoporous metamaterials and electrolytes under ultrafast pulsed laser excitation.

### 2.2 Photocurrent Generation Induced by NIR Ultrafast Laser Pulses

As a first step, we examined the charge emission process induced by plasmonic metamaterials when irradiated by an ultrafast laser source, focusing in particular on nanoporous gold. Specifically, we evaluated the opto‐electrical response of a thin porous gold layer. For this purpose, we fabricated porous passive MEAs, consisting of nanoporous gold electrodes used to measure photocurrent at the metamaterial/electrolyte interface.

The fabrication of nanoporous gold films can be considered a well‐established procedure and guarantees the easy integration of the material in MEA devices.^[^ ^29^ ^]^ The employed platform consists of a SiO_2_ substrate decorated with 24 planar porous gold electrodes. For clarity, Figure S1, Supporting Information depicts the fabrication process of an illustrative electrode. The device surface, except for the electrodes, is passivated with 2 µm thick SU‐8 for insulation during current measurements.

For measuring hot electron emission, we stimulated the nanoporous electrodes of the MEAs with a NIR ultrafast pulsed laser. In particular, we used a 1064 nm laser with 8‐picosecond long pulses repeated at 80MHz. Typically, we used trains of 8 ps pulses, with total duration of the pulse trains in the order of few milliseconds. The experimental setup was derived from the previous work of Zilio et al.^[^ ^27^ ^]^ and it is represented in Figure S2, Supporting Information.

**Figure 2:**
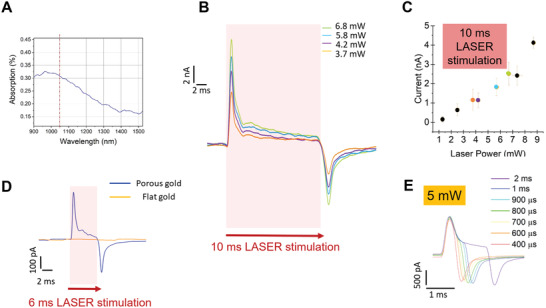
Photo‐generation of hot charges in nanoporous gold. A) Absorption profile of 50 nm thick porous gold. B) Current recorded with different laser intensity and with 10 ms pulse train duration. C) Faradaic photocurrent behavior with respect to the laser power. D) Comparison of the photocurrent recorded in the case of flat and porous gold surface when stimulated with 6 ms laser pulses. E) Recorded current in case of different laser pulses length and constant laser intensity of 5 mW.

As already mentioned in previous works,^[^ ^29^ ^]^ a thin layer of porous metal (50–200 nm pore size) behaves optically like a broadband absorber in NIR.^[^ ^35^, ^36^ ^]^ In **Figure** **2**A, we report the absorption spectrum of the nanoporous gold electrodes of the passive MEAs. We notice that the material has high absorption in the NIR range around 1 µm wavelength, which is ideal for the 1064 nm laser employed in our experiments.

We measured the generated current with a patch‐clamp amplifier in multiple sweeps of 250 ms each (for more details see Figure S2, Supporting Information and materials and methods section). We focused the laser spot on several positions over the available porous electrode surface in order to take into account topological variability. Each presented current profile represents in this way the average values obtained by mediating the recordings of the multiple sweeps on the same electrode with the same laser power.

Figure 2B–E shows the photo‐generated current at the metamaterial/phosphate‐buffered solution (PBS) interface as measured by the patch‐clamp amplifier. The current measurements show a sharp and fast initial positive peak at the beginning of excitation, a slowly decreasing positive shoulder during the laser pulse train, and a negative peak at the end of the laser pulse train. This particular behavior may be attributed to a combination of capacitive response and hot electrons emission of the system, resembling the current response obtained with other stimulation processes in which the capacitive response is coupled with a faradaic current.^[^ ^12^, ^37^, ^38^ ^]^ The initial current rise and the final negative peak are due to the capacitive charging and discharging process occurring at the electrode/electrolyte interface. Concurrently, for the whole duration of the laser pulse train, the hot electrons emission at the interface results in a positive sustained current flowing between the gold porous electrode and the saline solution.

We investigated the hot electron‐induced current with respect to the variation of laser power and pulse train duration. For each laser intensity, we measured the generated current with the amplifier in multiple sweeps of 250 ms each. We report an example in Figure 2B. In this configuration, we used a laser pulse train of 10 ms (8 ps laser pulses repeated at 80 MHz for 10 ms) for stimulating the electrode. We could notice an increase of both capacitive and faradaic components as the laser power rises. For a laser pulse duration of 10 ms, the faradaic photocurrent values are reported in Figure 2C. Notably, the hot electron current increases with respect to the impinging light power. We measured currents of hundreds of pA up to ≈5 nA for laser intensities ranging from 1 to 9 mW. For cellular photostimulation, we focused our attention in the range of currents from 1 to 3 nA obtained at laser powers from 3.71 up to 6.89 mW. In fact, at higher laser power levels, the photo‐generated current shows strongly irregular values due to the generation of cavitation bubbles at the electro/electrolyte interface, which change the efficiency of the hot electron formation and injection.^[^ ^27^ ^]^ Hence, for taking advance of plasmonically driven stimulation, the formation of these bubbles is undesirable. Moreover, the formation of these bubbles could be harmful for neighboring cells. Therefore, we keep the laser powers below this threshold.

In the evaluated power range, the recorded photocurrent is in the order of the nanoampere, which is compatible with the depolarization of the cellular membrane and thus with the stimulation of electrogenic cells.^[^ ^19^, ^37^ ^]^This characterization allowed us to better distinguish the optoporation process for intracellular recordings from that required for stimulation by metamaterials, as it will be described in the next section. In addition, we report in Figure S7, Supporting Information the photocurrent generated with other electrolytes typically used as cellular buffer solution such as artificial cerebrospinal fluid (aCSF) and Dulbecco's Modified Eagle Medium (DMEM). Here, we observe comparable current levels in the three considered electrolytes at various laser intensities.

For further assessing the contribution of the material morphology, we performed current measurements during laser excitation of flat and porous gold electrodes in parallel. In this case, we observed that the laser pulse train of the same intensity does not produce any detectable effect on flat gold surface in comparison with the porous counterpart (Figure 2D). This result is in line with previously reported control experiments involving the irradiation of cells cultured on flat and porous gold electrodes with ultrafast pulsed lasers.^[^ ^29^ ^]^ In this previous work, we assessed that the flat electrodes could not produce the plasmonic response necessary for cell optoporation. In accordance with previous works,^[^ ^27^ ^]^ the process generates only a negligible transient temperature raising at the picosecond regime, but it does not produce heat accumulation. To verify this, we investigated whether longer stimulation times would produce heating and thus affect the photocurrent. As represented in Figure 2E (laser intensity 5 mW), the value of the hot electron photocurrent does not increase or decrease while changing the pulse length, thus the duration of the laser pulse does not affect the amplitude of the photocurrent.

Moreover, from the measurements, we observed that there is no decrease in the efficiency of the system. Thus, since the electrode acts as a metal reservoir compensating the hot charges emission, the charges can be generated at each laser pulse and then accelerated and injected in the system without losing efficiency.^[^ ^27^ ^]^ This could be observed also in Figure S11, Supporting Information. In this experiment, we applied 10 and 100 ms laser pulses at 9 mW and we detected no change in the baseline in photocurrent, suggesting that no charge built‐up is occurring.^[^ ^39^ ^]^

These features point to consider the porous metamaterials as a valid alternative for highly effective and minimally invasive cellular stimulation.

### 2.3 Optical Stimulation of HL‐1 Cells on CMOS‐MEAs

To explore the applicability of the plasmonic stimulation, we used the immortalized cell line HL‐1^[^ ^40^ ^]^ (derived from mouse cardiac cells) and MEAs based on CMOS technology (CMOS‐MEA). The CMOS‐MEA platform provides already nanoporous platinum electrodes and offers very high spatial resolution for mapping precisely the propagation waves of both spontaneous and stimulated activity of electrogenic cells.

**Figure 3:**
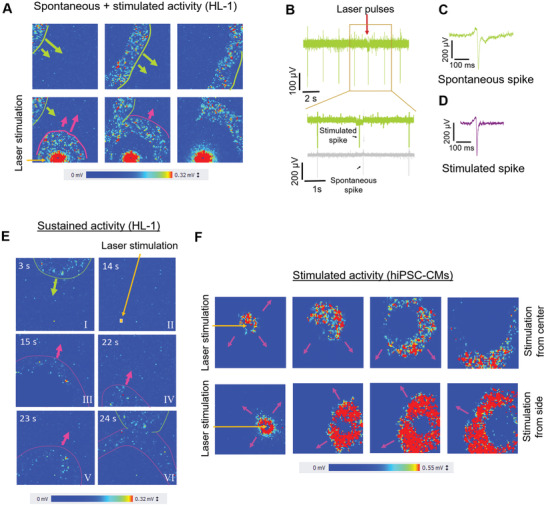
Optical stimulation of HL‐1 cells and hiPSC‐CMs. A) Instantaneous maps of spontaneous (highlighted in green) and stimulated activity (pink) propagation in HL‐1. The arrow indicates the propagation wave direction. B) Recording of the standard activity of HL‐1 before and after the stimulus application. In the inset, the stimulated field potential event compared with the physiological one. C) Magnification of the field potential generated in standard condition. D) Magnification of the stimulated field potential. E) Example of the persistent pattern induced by the optical stimulation. F) Instantaneous maps of stimulated activity propagation in hiPSC‐CMs. Please refer to the corresponding movies in the Supporting Information for further details and clarifications, as indicated in the main text.

In physiological conditions and without optical stimulation, we observe a regular electrical activity with typical waves propagating in defined directions (**Figure** **3**A, top row). Specifically, three snapshots of the CMOS‐MEA array show the propagation of an activity waveform coming from the top‐left corner. Each pixel is representative of a single electrode, the colors range from blue to red accordingly to the amplitude of the recorded potential variation.

Then, we focus the laser on a selected electrode for stimulating the HL‐1 cells with a 1064 nm laser pulse train and an impinging power ranging from 4 to 7 mW (Figure S3, Supporting Information). We focus the laser spot on the surface of the nanoporous metamaterial, at the interface between the electrode and the cellular membrane. Immediately following laser irradiation of the metamaterial electrode, we observe the generation of single propagation waves originating from the stimulated cell, in addition to the spontaneous propagation wave already present in the culture. An example is shown as time snapshots in Figure 3A, bottom row. The green highlighted wave from the top‐left corner is the original propagation pattern of the culture, whereas the pink highlighted wave is a secondary propagation wave that originates from the stimulated cell at the bottom of the map (blue square) immediately after laser irradiation. The red colored region around the stimulated electrode is due to the saturation (toward positive voltage) of the surrounding electrodes, which are temporary affected by the laser excitation, as we have shown in previous work.^[^ ^29^ ^]^ This effect is related to electrical noise of the amplifiers of the CMOS‐MEA and it does not produce any effect on the surrounding cells.

(For the whole recording and movies of other examples, see Movies S1 and Movie S2, Supporting Information). In Figure 3B–D, we report exemplary traces of spontaneous and stimulated field potentials of HL‐1 cells. In Figure 3B, the green time‐trace represents the activity of a cell that is stimulated with laser at the time indicated by the red arrow. In fact, we can observe that the fifth field potential occurs slightly earlier than expected by the spontaneous beating rate. The inset in Figure 3B clearly shows this temporal displacement of the stimulated field potential (green) by comparing it to spontaneous signals (gray) in physiological condition.

Figure 3C reports an enlarged view of a field potential belonging to the stimulated wave that propagates from the bottom electrode in Figure 3A. The shape of the stimulated field potential is fully comparable with that of spontaneous activity (Figure 3D). The CMOS MEAs allow us also to calculate the propagation velocity of cardiac activity by measuring the time difference on the field potentials of cardiomyocytes on electrodes at defined distances. The resulting velocity is 20 ± 2 mm s^−1^, which is comparable to values measured for the same cellular model in other works^[^ ^41^ ^]^. Notably, the velocity of propagation of the stimulated wave is in the same range of the physiological wave (18 ± 3 mm s^−1^).

Next, we verified that the laser stimulation does not affect negatively the cells and does not lead to cell death. We observe that the physiological activity is preserved also after the application of the laser stimulus (for long recording please refer to Figure S4, Supporting Information). In fact, the beating rate calculated before and after the stimulation are, respectively, 25.1 ± 0.1 and 25.0 ± 0.1 bpm. Thus, the stimulation does not influence the physiological activity of the culture.

Interestingly, in few cases, we were also able to create a temporary persistent alternative propagation pattern, which is repeated more than once after a single laser stimulation. We display an example in Figure 3E, where we report six snapshots from a recording of the 4096 electrodes before and after the stimulation. From *t* = 0 s to *t* = 13 s, we observe the original propagation wave starting from the top and moving downward (panel I). At *t* = 14 s, we performed laser stimulation on the electrode highlighted with a yellow square (panel II). As expected, we observe a stimulated propagation wave starting from this electrode (panel III). However, from *t* = 22 s to *t* = 24 s, we observe that there are still two different propagation patterns running together, the original wave from the top (green) and an alternative wave (pink) starting from the stimulated cell and moving upward (panels IV, V, VI). The newly generated pattern lasts for few cycles (2–3 cycles), then the cell monolayer restores the original physiological activity, with only the downward propagation pattern from the top. Although the mechanism behind the optically generation of persistent propagation waves requires a deeper biological investigation, it shows high potential for altering and controlling activity in cardiac tissues (For the whole recording refer to Movie S3, Supporting Information). We report exemplary recordings of spontaneous and sustained field potentials in Figure S5, Supporting Information (extracted from the activity shown in Figure 3E).

In addition, in Movie S5 and Figure S10, Supporting Information, we report a video that shows the possibility of triggering a spiral wave‐front with our system. This peculiar reentrant electrical activation is typical for common cardiac arrhythmia events.^[^ ^42^, ^43^ ^]^ Under further exploration, this property could strengthen the use of plasmonic‐mediated stimulation as a tool to develop safe and effective assays for cardiac arrhythmias.

In summary, with the presented configuration, we observe that the hot electron current produced by excitation of nanoporous electrodes with pulsed laser can elicit action potentials in HL‐1 cells at a laser power of ≈5 mW. This therefore suggests that optical stimulation of cardiac cells is feasible using plasmonic metamaterials irradiated with ultrafast pulsed laser directly on CMOS MEAs. In total, we performed experiments on 20 HL‐1 cells distributed on 6 CMOS‐MEAs over 3 cell preparations.

At laser powers below 5 mW, the stimulation success rate decreases rapidly, with no occurrences of stimulation at powers of ≈1 mW. This trend is in line with the observed decrease of photocurrent generated at lower laser intensities, as displayed by the patch‐clamp measurements in the previous section. In particular, the faradaic component of the photo‐generated current decreases by almost one order of magnitude from 5 to 1 mW laser power.

Such threshold behavior in the required laser intensity renders the stimulation technique complementary to the process of optoacoustic cell poration and intracellular recording, which occurs at laser powers in the range of 1 mW on the same nanoporous materials (gold and platinum).^[^ ^29^ ^]^ In fact, as in the case of electroporation and electrical stimulation achieved on the same device, we have identified this threshold of laser intensity as the limit for which the optoacoustic poration occurs without stimulation. Therefore, by tuning the employed laser power, one can obtain only intracellular recording or intracellular recording and stimulation on the same MEA device.

Due to the tight adhesion of cells on the nanoporous metamaterials around the laser stimulation site, the nanopores in the cellular membrane are insulated in a confined compartment and do not lead to leakage or ionic exchange with the extracellular medium.^[^ ^29^ ^]^ This ensures that the poration mechanism does not interfere with activity stimulation by ultrafast pulsed laser irradiation. In fact, the insulation of membrane nanopores due to tight sealing ensures that the resting membrane potential of the stimulated cell is not affected by poration. Experimental results from previous works^[^ ^29^, ^30^ ^]^ on porated cardiomyocytes support this claim, because porated cells do not show alteration of their spontaneous electrophysiological activity. As we show in Section 2.5, stimulation experiments on primary neurons also support this statement. The confinement of the generated photocurrent and of the resulting redox reactions also ensures that the effect on cells is maximized. In fact, the confinement avoids the dispersion of the produced redox reactions into the cellular medium.

### 2.4 Optical Stimulation of Human‐Derived Cardiomyocytes on CMOS‐MEAs

We further verified the efficacy of the methodology with human cardiomyocytes derived from induced Pluripotent Stem Cells (hiPSC‐CM) (Cellular Dynamics International), which are a far more relevant biological model than HL‐1 cells. The higher biological relevance of hiPSC‐CMs stems from the fact that these cells present a complex variety of ion channels, which makes them respond to drugs in a way similar to how human cardiomyocytes do. On the contrary, HL‐1 cells present a simpler ion channel configuration and cannot be used in pharmacological screenings. We cultured the hiPSC‐CMs in 2D syncytia on CMOS‐MEAs and incubated the samples until they presented a spontaneous mechanical contraction and a concomitant electrical activity.

As for HL‐1 cells, we recorded first the spontaneous electrophysiological activity for few minutes before starting with the stimulation procedure. For stimulation, we used laser power of ≈5 mW as in the case of HL‐1 cells. As shown in the snapshots in Figure 3F, laser irradiation of the metamaterial electrodes elicits propagation waves that originate from the stimulated cardiomyocyte (full video available as Movie S4, Supporting Information). Notably, we are able to subsequently stimulate different cells within the same cell cultures (example depicted in the two sequences in Figure 3F). After the optical stimulation of new propagation waves, the hiPSC‐CMs always recovered their spontaneous electrophysiological activity. In fact, the hiPSC‐CM cultures were placed again in the incubator after the stimulation procedure, and their activity was tested again up to 2 days later without observing variations in respect to the original physiological activity.

Furthermore, thanks to the high resolution of the CMOS‐MEAs, we could also easily evaluate the propagation velocity of the physiological and stimulated waves. The two resulting velocities do not present statistically significant differences and are respectively 22 ± 2 and 19 ± 1 cm s^−1^. Moreover these two values are comparable to those typically observed for this cellular model.^[^ ^44^, ^45^ ^]^ Thus, the methodology allows the concurrent evaluation of the beating rate, velocity, and pattern propagation of spontaneous and stimulated waves with high spatial and temporal precision. In total, we performed successful stimulation of hiPSC‐CMs on 4 CMOS‐MEAs distributed over two distinct cell preparations.

### 2.5 Optical Stimulation of Primary Neurons

**Figure 4:**
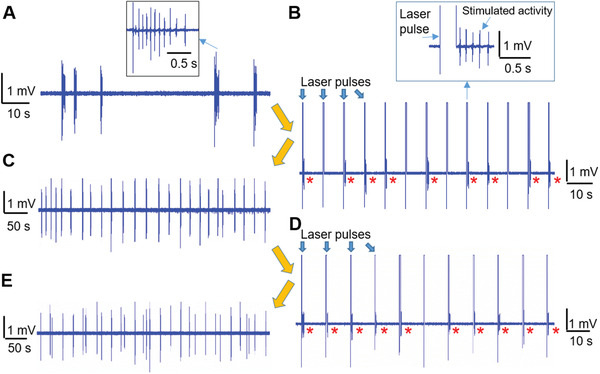
Optical stimulation of rat primary neurons. A) Spontaneous bursting activity of rat primary neurons on CMOS‐MEA. B) Burst activity on the same electrode stimulated by periodic laser pulses (8 s repetition period). The red asterisks indicate the presence of stimulated activity after the laser pulse. The inset shows a magnified view of a laser stimulation and consequent stimulated burst activity. C) Recovered spontaneous bursting activity after stopping the laser stimulation. D) A second series of periodic stimulation with 10 s repetition period. E) Recovered spontaneous bursting activity after stopping the second series of laser stimulation.

In a further series of experiments, we have tested the performance of our approach for stimulating electrophysiological activity in hippocampal primary neurons from rats. Here, we cultured neurons on CMOS‐MEAs and incubated the cells for 21 days in vitro (DIV), obtaining mature and active neuronal networks (details of the culture protocols are in the materials and methods section). In **Figure** **4**A, we report an exemplary recording from an electrode of a CMOS‐MEA, showing spontaneous burst activity as expected for mature neuronal networks.^[^ ^46^ ^]^ On the same electrode, we apply multiple laser pulses with 4 mW power and a constant repetition period of 8 s, while continuously recording the neuronal activity. The trace in Figure 4B shows the recording, where the laser pulses can be recognized as positive saturation peaks of the signal at 4 mV (upper limit of the CMOS‐MEA recording device). The red asterisks indicate all occurrences of neuronal activity immediately after the laser pulse and thus optically stimulated, as depicted in the magnified inset, where we can clearly see the train of spikes following the laser pulse stimulation. As expected for neurons connected in a mature network, the stimulated activity propagates to the rest of the culture, which shows bursting activity synchronized with the firing of the stimulated neuron (Figure S6, Supporting Information). Interestingly, we observe that the neuronal activity locks almost completely to the periodicity of the laser stimulation, without any spontaneous activity recorded outside of the laser stimuli. The absence of prolonged firing from the stimulated neurons confirms that cell stimulation is not related to ion leakage and variations of the resting membrane potential, which would cause repeated firing due to continuous depolarization of the affected cells.

After a total stimulation time of 4 min, we stop the optical stimulation and continue to record the neuronal activity for evaluating the recovery of the spontaneous signaling (Figure 4C). We observe that the burst activity quickly loses the 8 s periodicity and settles at an interburst interval (IBI) of 19.14 ± 7.2 s. Subsequently, we stimulate again the same electrode with a different periodicity (10 s), observing that the neuronal activity locks again to the new (different) stimulation pattern (Figure 4D). Remarkably, at the end of the stimulation protocol, the activity acquires once again a typical spontaneous bursting activity with IBI of 19.7 ± 8.2 s, thus very similar to the IBI value before the last stimulation sequence. This experiment strongly supports the very low invasiveness of the stimulation protocol, as confirmed by the fact that the process can be repeated on the same neuron without negative consequences on the basal activity in absence of stimulation.

The traces in Figure 4 are reported in Figure S8, Supporting Information with different time scales for a clearer comparison of the burst duration. We have calculated the burst durations in the different phases of the experiment. As shown in Figure S9, Supporting Information, the stimulated bursts present a shorter duration of ≈400 ms in respect to the spontaneous bursts (≈1 s). The change of duration may be attributed to the different nature of the bursts. In fact, the spontaneous bursts are related to the overall activity of the whole network, whereas the stimulated activity stems from the elicitation of a single neuron.

Overall, we performed successful stimulation experiments on 12 neurons distributed on 6 CMOS‐MEAs over 3 distinct neuronal preparations. We calculated an average stimulation success rate of 68% ± 10%, intended as the probability that a laser excitation event results in the stimulation of a spike or burst. During our experiments, we observed that stimulation of neurons could be reliably achieved by exciting with laser the neuronal somas. When pointing the laser on axons or neuronal processes, we could not obtain reliable stimulation of neurons. We suggest that this difference stems from the different adhesion of somas and processes on the CMOS‐MEA, whose electrodes are embedded 2 µm below the passivation layer and thus create a nonflat surface.

## 3 Conclusion

We present experimental data that demonstrate the capability of plasmonic metamaterials to elicit action potentials in cardiac and neuronal cells under ultrafast pulsed laser illumination, without genetic modifications of the cells and without administration of optically active nanomaterials.

The optical stimulation stems from a hot electron emission process activated by photoexcitation of porous plasmonic metamaterials with ultrafast NIR laser pulses. We experimentally observed the charge injection by measuring the electric current flowing from porous electrodes into an electrolyte solution. We found that the photo‐generated currents are compatible with cellular activity stimulation.

Remarkably, the exploitation of porous materials enables the cell stimulation directly on the electrodes of high‐resolution CMOS‐MEA devices, providing at the same time localized stimulation and concurrent electrophysiological recording of large cellular ensembles.

To show the efficiency and the advantages of the mechanism, we successfully stimulated electrophysiological activity in HL‐1 cells, hiPSC‐CMs, and rat primary neurons directly on CMOS‐MEAs with porous platinum electrodes. In HL‐1 cells and human‐derived cardiomyocytes, the laser excitation has demonstrated to stimulate secondary propagation patterns in addition to the spontaneous activity of the culture. Moreover, in some cases, the stimulation resulted in the temporary promotion of the stimulated cell to the role of pacemaker, thus creating new pathways of artificial propagation. The successful stimulation and recording of hiPSC‐CMs is particularly promising application wise, since these cells represent a very reliable model for drug discovery, screenings, and cardiac toxicology. This approach therefore offers the possibility to use the single‐cell stimulation technique on high‐density and high‐resolution sensors, paving the way for new methodologies for the study of cardiomyocytes and cardiac tissues. Indeed, the integration of cell stimulation technologies during screening assays should lead to improvements in drug discovery.^[^ ^9^ ^]^ In particular, the variation of the cell pacing during the same experiment could help validate drug's efficacy or toxicological effects with respect to the whole spectrum of heart beating rate.

In rat primary neurons, we were able to stimulate mature neuronal networks cultured on CMOS‐MEAs, while simultaneously recording their activity on the network level. In particular, we could lock the neuronal activity to periodic laser stimulations, achieving an almost one‐to‐one relation between laser stimulation and burst activity. Remarkably, we did not observe negative effects of the laser stimulation on the neuronal cultures, which could recover their basal electrophysiological activity after multiple stimulation protocols.

Additionally, since optical stimulation is concurrent with optoporation, intracellular recordings and AP stimulation can be obtained simultaneously, providing an overall view of the culture activity and a highly resolved evaluation of drug effects on cellular ion channels. This combination could thus provide a powerful noninvasive way of evaluating cell behavior at multiple levels.

It is significant that the presented technique is compatible with electrophysiology platforms already present on the market. This can facilitate the applicability of the methodology in various research fields. As an example, external stimulation has been demonstrated to cause differentiation in hiPSC.^[^ ^47^ ^]^ Hence, this technology holds high potential also for tissue engineering and patient‐specific applications. Therefore, through future developments, the sensing and stimulation capabilities of plasmonic metamaterials may lead to integrated multimodal systems for cellular analyses and stimulation.

Furthermore, plasmonic metamaterials may also open the possibility of optical stimulation in in vivo applications. In the first place, the porous materials could be easily fabricated and integrated on flexible and biocompatible substrates suitable for in vivo applications, for regenerative medicine or tissue engineering applications. Moreover, this technology offers high spatio‐temporal control together with remote access to optical pacing. This, together with operation at NIR wavelength, makes the technique particularly favorable for these applications, owing to reduced photon scattering and absorption in thick tissues and at the same time avoiding damages at the cellular level while providing deeper penetration. These last aspects may form the basis for the new generation of all‐optical plasmonic pacemakers and muscle actuators that provide both low invasiveness and no cellular type restrictions.

## 4 Experimental Section

### Porous MEA Fabrication

Following standard MEA fabrication steps, pads were designed through standard photolithography technique already developed in previous work. In detail, a first layer of LOR3B (MicroChem) was used as undercut layer and was spin coated on the quartz wafer and baked at 180 °C for 5 min. A second layer of 2  µm of S1813 (Microposit) was subsequently spin coated on top of the previous layer and baked at 90 °C for another 5 min. The 24 electrodes were defined with UV exposition for 12 s and after the development in MF‐319 (Microposit Developer) for 60 s. A 5 nm titanium adhesion layer and a 10 nm gold layer were deposited by means of electron‐beam evaporation in a high vacuum chamber at a 0.3 Å s/1 deposition rate. The alloy of silver/gold was then sputtered on the gold layer and after lift‐off process in hot remover PG (MicroChem) the definition of the final MEA design was completed. The wafer was then rinsed out in acetone and isopropanol, and the dried with nitrogen. The wafer was at this point cut and each MEA device was separated. The so‐constituted MEAs were immersed in nitric acid solution in which silver was etched away selectively, resulting in highly porous gold structures. At this stage, the device presented multiple electrodes arranged in a 6 × 4 array with inter‐electrode distance of 400 µm. After cleaning the sample, the whole surface except for the electrodes and the external pads, was passivated with 1.5 µm SU‐8 in order to avoid leakage while the device will be in solution. After a hard baking at 180 °C, the sample was mounted on a printed circuit board (PCB) and passivated with epoxy resin that covered the electrical connections with the PCB and allowed a glass ring to be fixed on the surface to form a culture well.

### System for Photocurrent Measurements

The laser was focused onto the porous electrodes by means of a 60 × immersion objective (NA = 1) using a CCD camera Nikon DS‐Fi2 for visualizing the electrodes. The porous MEA electrode was immersed in PBS and its external pad was electrically connected to pre amplifier (CV‐7B headstage) and amplifier (Axopatch 200B for ultralow noise single‐channel recordings) in order to record the charge injection. The ADC (Axon Digidata 1550B plus HumSilence) was then connected to the amplifier in order to both send TTL signals for light pulse control and to interface the amplifier and the computer.

A rotating polarizer was used to change the laser intensity while the current at the electrode–electrolyte interface is measured. The laser intensity was measured at the beginning of each experiment by means of a power meter situated at the working distance of the objective.

A platinum wire immersed in the solution acted as a counter‐electrode for the current measurements; all measurements of photocurrent were made without the application of a bias between the platinum counter‐electrode and the sample. The measurements were performed in voltage clamp mode (set in the amplifier).

### Laser Stimulation and Photocurrent Generation Setup

The 1064 nm (Nd:YAG [neodymium:yttrium–aluminum–garnet] solid‐state laser (Plecter Duo [Coherent]) was used as the light source, for which the emission is in ultra‐short pulses at 8 ps with 80 MHz repetition rate. The pulsed beam was then switched ON and OFF at the desired pulse length, generating pulse trains ranging from microseconds to hundreds of milliseconds.

Throughout the manuscript, the term pulse length referred to the ON time of the 8 ps pulsed laser, defined with an acousto‐optic modulator (AOM) or a mechanical shutter controlled by a TTL signal from the analog‐to‐digital signal converters (ADC/DAC Axon Digidata 1550B plus HumSilencer) connected to the software (Axon pCLAMP).

The laser was combined to an upright microscope (Ecplise FN‐1 from Nikon) able to accommodate the BioCAM acquisition system from 3Brain GmbH directly on the microscope stage. A 60 × water‐immersion objective (NA 1.0) was inserted in the cell medium or PBS during the experiment in order to focus the NIR laser used for stimulation. For the purpose, the porous MEA electrodes were immersed in PBS (alternatively in aCSF or DMEM) as well, and connected with the amplifier (Axopatch 200B), as displayed in Figure S2, Supporting Information.

### HL‐1 Cell Culture

CMOS‐MEAs were sterilized with 20 min UV exposure in laminar flow hood. HL‐1 cell derived from adult female C57BL/6J mouse line were used and acquired from Sigma‐Aldrich (SCC065). To improve the cell adhesion, the devices were treated with poly‐l‐lysine (Sigma‐Aldrich) for 5 min, washed extensively with water, and let dry in sterile condition. Next, HL1 cells were seeded at the density of 84 000 per cm^2^ and grown with Claycomb culture medium, supplemented with 10% fetal bovine serum, 100 µm norepinephrine, 300 µm ascorbic acid, 2 mm l‐glutamine and penicillin and streptomycin (Sigma‐Aldrich). The culture medium was changed every day. When 100% confluence was reached (after 5 days), the electrical activity of cells was monitored with the BioCAM acquisition system from 3Brain AG.

### hiPSC‐CM Culture

Human iPSC‐derived cardiomyocytes (hiPSC‐CMs) were purchased from Cellular Dynamics International. iCell cardiomyocytes, a mix of ventricular, atrial, and nodal‐like cells, were seeded on commercial high‐density CMOS‐MEAs available from 3Brain AG. CMOS‐MEAs were previously sterilized using 70% ethanol for 30 min and then coated with fibronectin (Corning) for 1 h at 37 °C, 5% CO_2_ in a humidified incubator. Thereafter, the coating solution was aspirated and hiPSC‐CMs were rapidly plated at a density of 18 000 cells per CMOS‐MEA. Electrophysiological recordings were performed starting from 14 days post‐plating according to the specifications of the commercial supplier.

### Rat Primary Neurons Culture

CMOS‐MEAs were sterilized with ethanol 70% in water for 20 min, washed with sterile water three times, and dried in sterile conditions for at least 2 h. After that, the devices were pre‐conditioned by overnight incubation at 37 °C, 5% CO_2_, and 95% humidity in primary neural growth medium (PNGM), supplemented with 2 mm l‐glutamine, GA‐1000, and 2% neural serum factor‐1. The following day, the PNGM was removed from the culture well to coat it with a solution of 30 µg mL^−1^ poly‐d‐lysine (Sigma‐Aldrich) and 2 µg mL^−1^ laminin (Sigma‐Aldrich) in PBS in order to enhance primary neuronal cells adhesion and proliferation on the devices.

After coating, the device was again incubated for 4 h at 37 °C, 5% CO_2_, and 95% of humidity. After that, the substrates were washed extensively with sterile water four times and dried in sterile conditions overnight before cell seeding. Neuronal cells (Lonza Walkersville, United States) were seeded on the devices (2 × 10^5^ cells per device) and incubated at 37 °C, 5% CO_2_, and 95% humidity. Upon adhesion, after 2–2.5 h, PGNM was partially removed, leaving a small volume to ensure the cells do not dry, and fresh medium was added. Cultures were maintained for more than 3 weeks, while every 3–4 days, half of the medium was changed with fresh PNGM.

### Optical Stimulation Procedure

During the optical stimulation protocol, the cardiac cells were cultured directly on the CMOS‐MEAs and incubated for 4–5 DIV until they reached confluence. Using the same optical setup presented in previous works^[^ ^29^ ^]^, the laser was coupled to an upright microscope and with the BioCAM acquisition system placed directly on the microscope stage (see Figure S3, Supporting Information). A 60 × water‐immersion objective (with NA = 1.0) was inserted in the cell medium during the experiment in order to observe the cells on the CMOS‐MEA electrodes and to focus the NIR laser used for stimulation.

At first, the physiological extracellular activity was recorded for about 10 min in order to characterize the culture. Second, laser pulse protocol was applied on the porous platinum meta‐electrodes of the CMOS‐MEA and were used to induce the cellular stimulation. Typically, a laser power of ≈5 mW was used.

### Optical Characterization

The optical characterization of the porous gold was performed by using a custom‐made optical setup for transmittance and reflectance measurements at normal incidence. The system was equipped with an Ocean Optics NIRQUEST spectrometer, that allowed for measuring optical spectra in the NIR range (850–2500 nm). Reflectance spectrum of porous gold was measured in respect to a thick, perfectly reflective gold layer. Transmittance spectrum of porous gold was measured with respect to a quartz substrate. Successively, the absorption spectrum of porous gold was calculated as A = 1 – (T + R), neglecting in a first approximation the scattering contribution.

## Conflict of Interest

The authors declare no conflict of interest.

## Author Contributions

The manuscript was written through contributions of all authors. M.D. and F.D. conceived and planned the experiments. G.B., G.M., G.I., M.P., and R.M. carried out the sample preparation and experiments. G.M. and G.I. performed the cell cultures. A.B. performed the optical characterization. All authors have given approval to the final version of the manuscript.

## Supporting information

Supporting Information

Supplemental Movie 1

Supplemental Movie 2

Supplemental Movie 3

Supplemental Movie 4

Supplemental Movie 5

## Data Availability

The data that support the findings of this study are available from the corresponding author upon reasonable request.
