# Supplementary material for: All‐Optical and Label‐Free Stimulation of Action Potentials in Neurons and Cardiomyocytes by Plasmonic Porous Metamaterials
Source: Adv Sci (Weinh). 2021 Sep 5;8(21):2100627. doi: 10.1002/advs.202100627 (PMC8564419; doi:10.1002/advs.202100627)
Supplement: Supplementary file 1 — Supporting Information [file ADVS-8-2100627-s003.pdf]

© 2021 Wiley-VCH GmbH

# Supporting Information

for *Adv. Sci.,* DOI: 10.1002/advs.202100627

# All-optical and label-free stimulation of action potentials in neurons and cardiomyocytes by plasmonic porous metamaterials

*Giulia Bruno, Giovanni Melle, Andrea Barbaglia, Giuseppina Iachetta, Rustamzhon Melikov Michela Perrone, Michele Dipalo* and Francesco De Angelis**

**Supporting Information**

# All-optical and label-free stimulation of action potentials in neurons and cardiomyocytes by plasmonic porous metamaterials

Giulia Bruno, Giovanni Melle, Andrea Barbaglia, Giuseppina Iachetta, Rustamzhon Melikov, Michela Perrone, Michele Dipalo* and Francesco De Angelis*

1. Fabrication process of porous gold Multielectrode Array ........ 3
2. Characterization of photocurrent optical setup ........ 4
3. Optical stimulation optical setup ........ 5
4. HL1 long recording after stimulation ........ 6
5. Temporary persistent propagation pattern recording ........ 7
6. Neuronal synchronized bursting activity ........ 8
7. Photocurrent generated at electrode-electrolyte interface in the case of most used cellular medium 9
8. Stimulation of neurons ........ 10
9. Analysis of spontaneous and stimulated burst activity ........ 11
10. Instantaneous frames of spontaneous activity and stimulated spiral wave-front ........ 12
11. Long-term photocurrent measurements with multiple events ........ 13

1. Fabrication process of porous gold Multielectrode Array

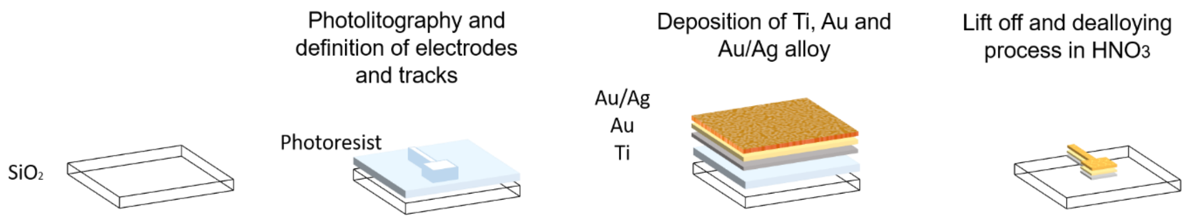


**Figure S1.** Schematic representation of the porous gold electrodes and its fabrication steps

2. Characterization of photocurrent optical setup

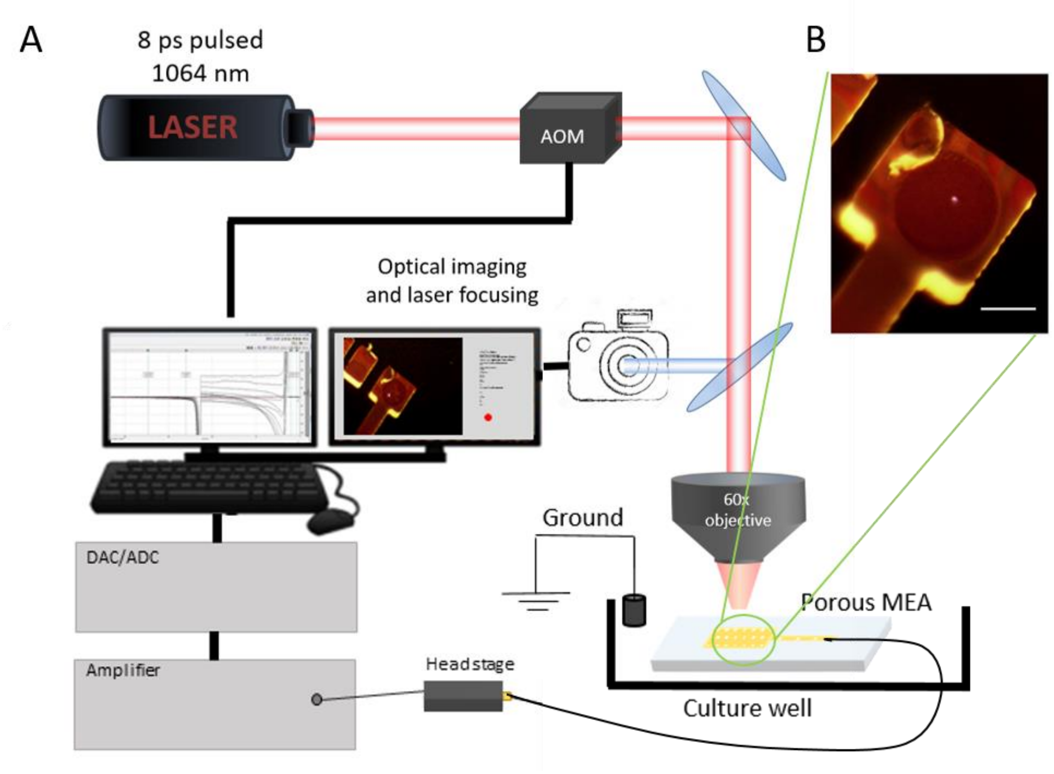


**Figure S2.** A) Optical setup composed of ultra-fast NIR laser, the modulator and objective which focuses light onto the electrodes. The recording is performed through patch clamp amplifier and ADC connecting MEA electrodes. B) Images from the CCD camera connected to the microscope capturing the laser spot on porous electrode. Scale bar 10 µm

3. Optical stimulation optical setup

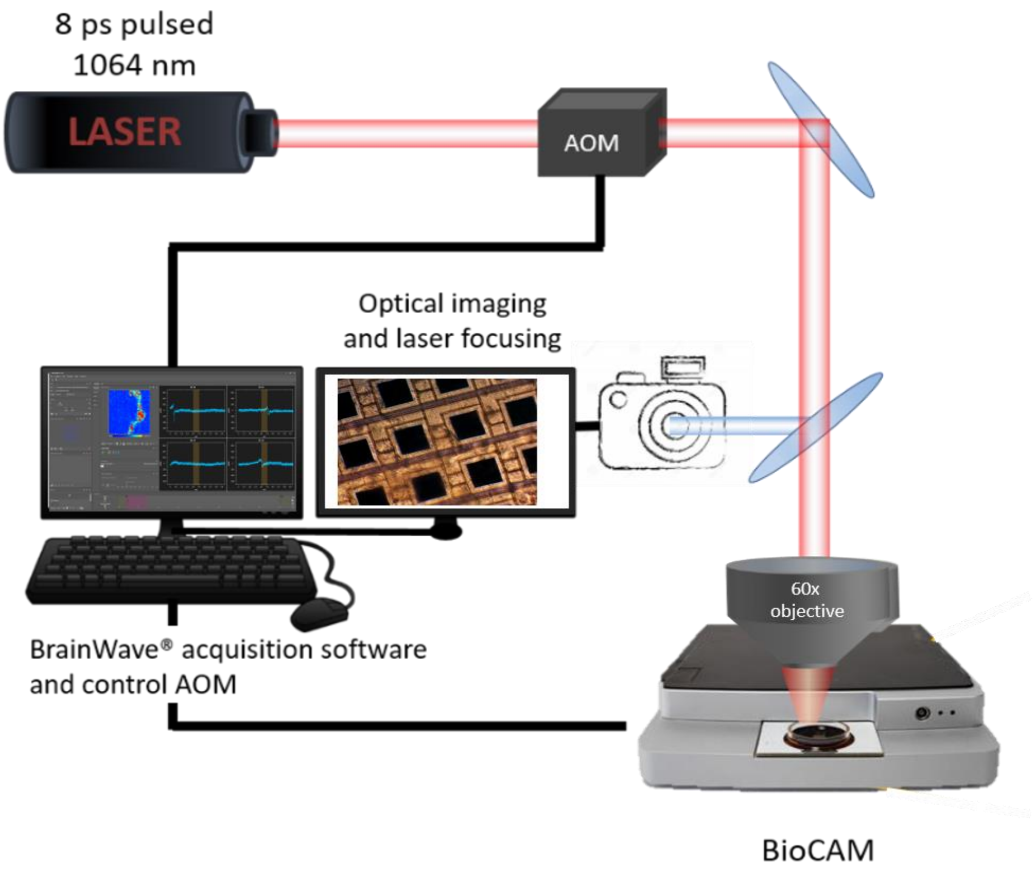


**Figure S3**. Optical setup integrated with BioCAM acquisition system.

4. HL1 long recording after stimulation

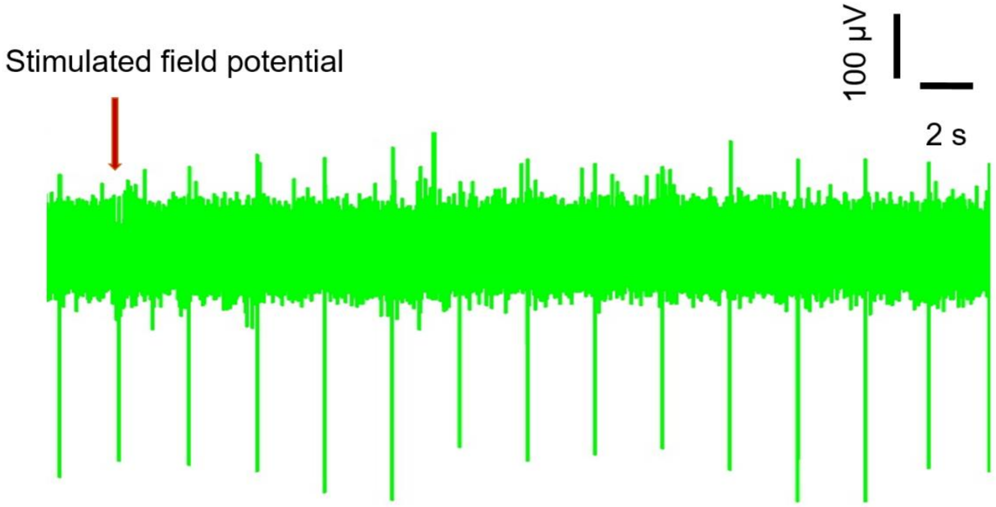


**Figure S4**. A) Long recording of the HL1 activity after the stimulated event indicated by the red arrow.

## 5. Temporary persistent propagation pattern recording

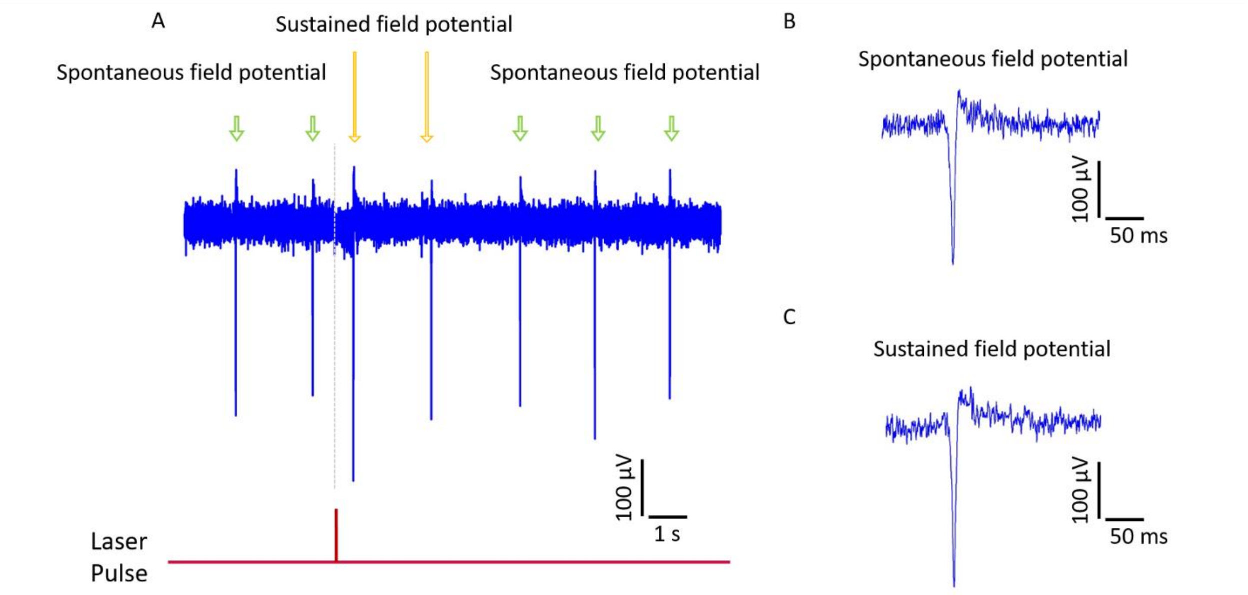


**Figure S5**. A) Recording of the standard + sustained activity of HL-1 before and after the stimulus application. B) Magnification of the spontaneous field potential event. C) Magnification of the sustained field potential for comparison.

6. Neuronal synchronized bursting activity

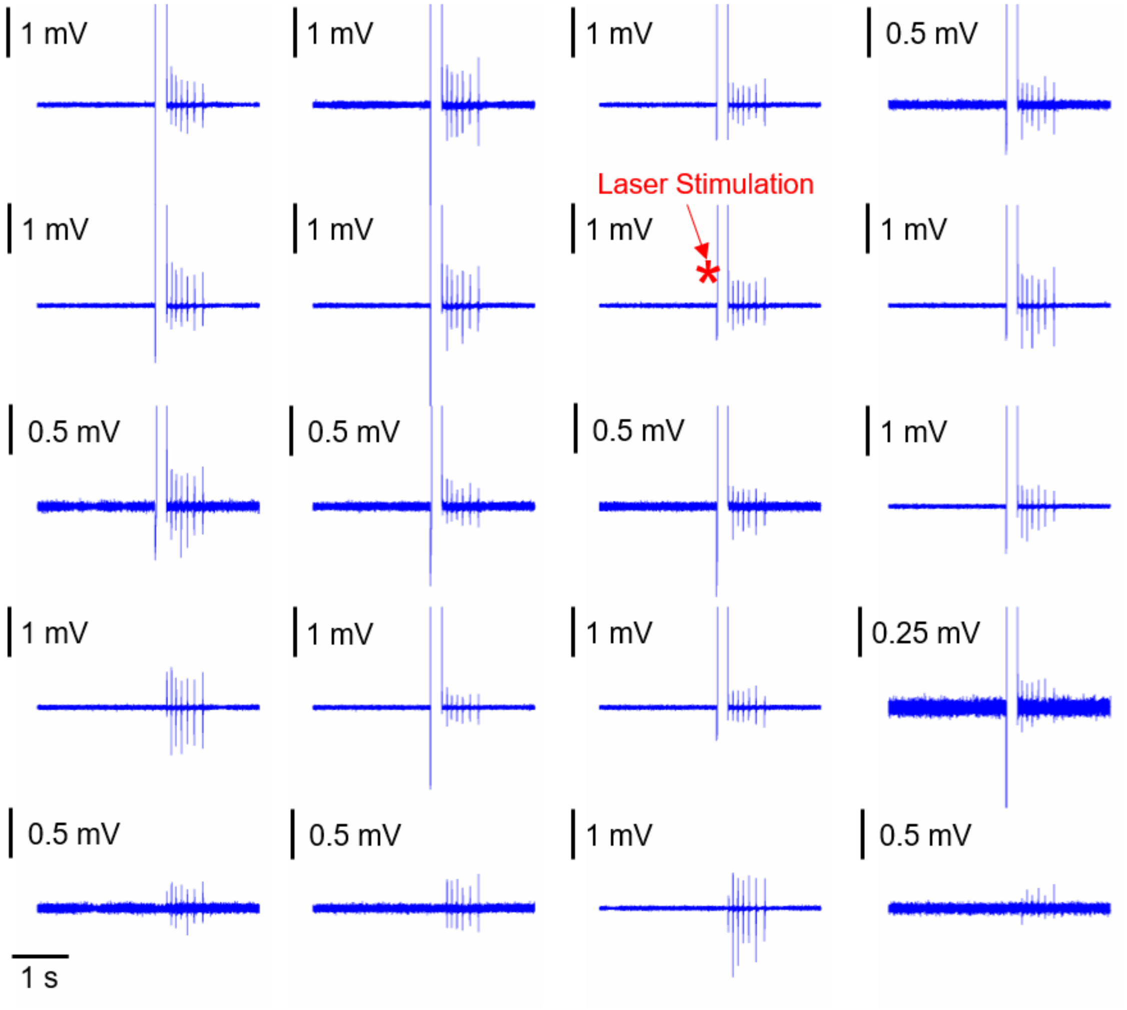


**Figure S6.** A) Recordings of stimulated neuronal bursting activity of rat primary neurons from multiple electrodes on CMOS-MEA. The trace with the red asterisk represents the electrode on which the laser stimulation has been applied. The activity on the surrounding electrodes confirms that the stimulated activity propagates through the neuronal network.

7. Photocurrent generated at electrode-electrolyte interface in the case of most used cellular medium

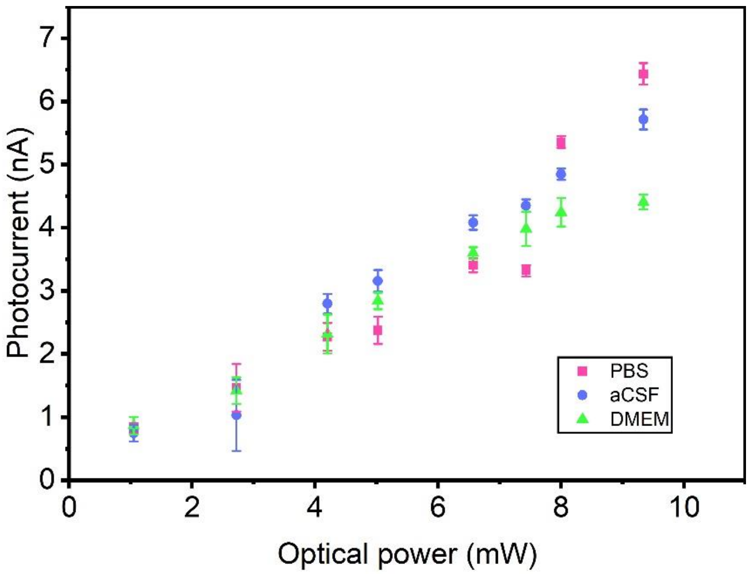


**Figure S7**. Photocurrent generated at the gold electrode-electrolyte interface using aCSF and DMEM solution as electrolytes and compared with PBS.

## 8. Stimulation of neurons

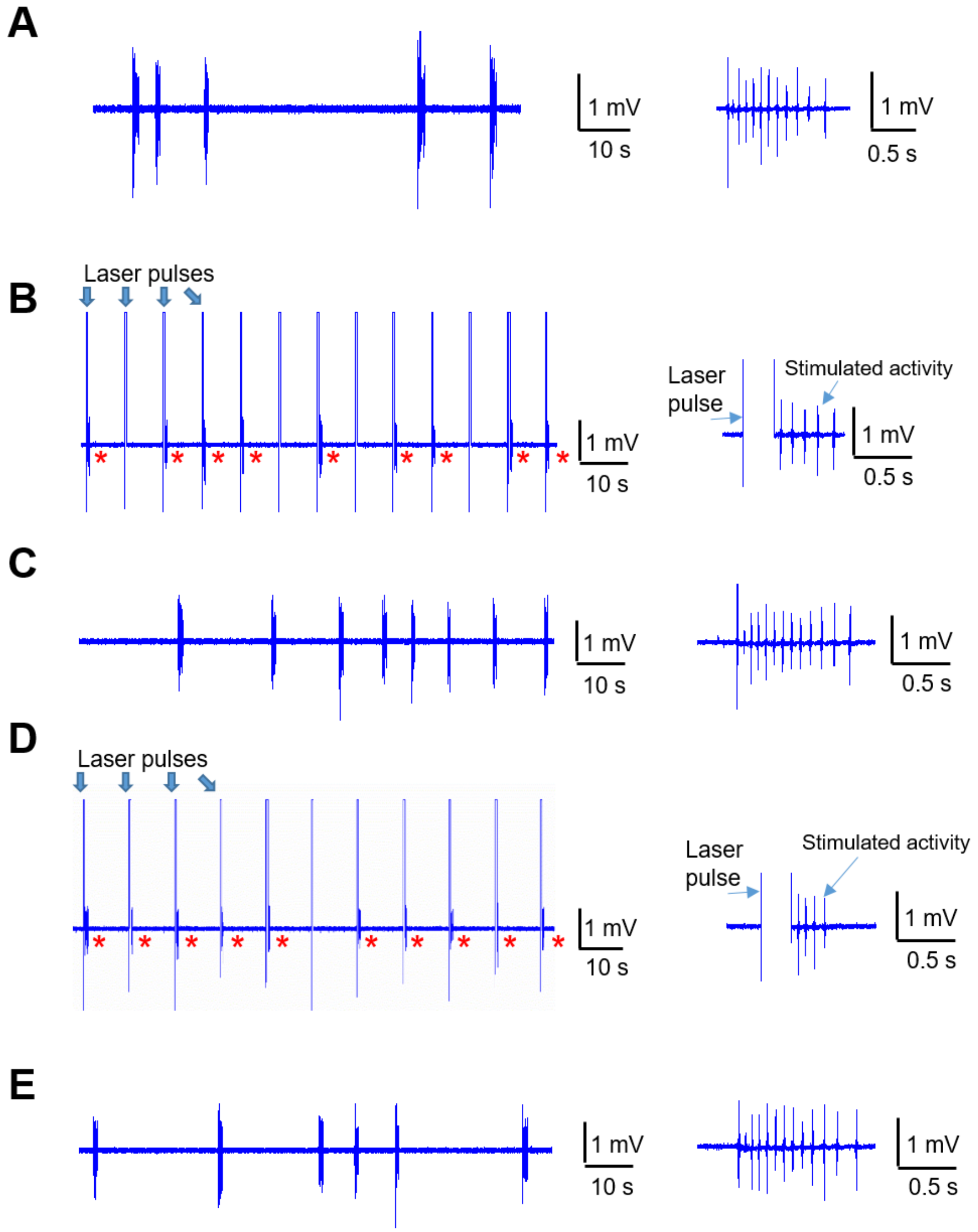


**Figure S8**. A-E) Magnified views of the spontaneous and stimulated neuronal activity from figure 4 of the manuscript. On the right side, magnified views of neuronal bursts for each case.

9. Analysis of spontaneous and stimulated burst activity

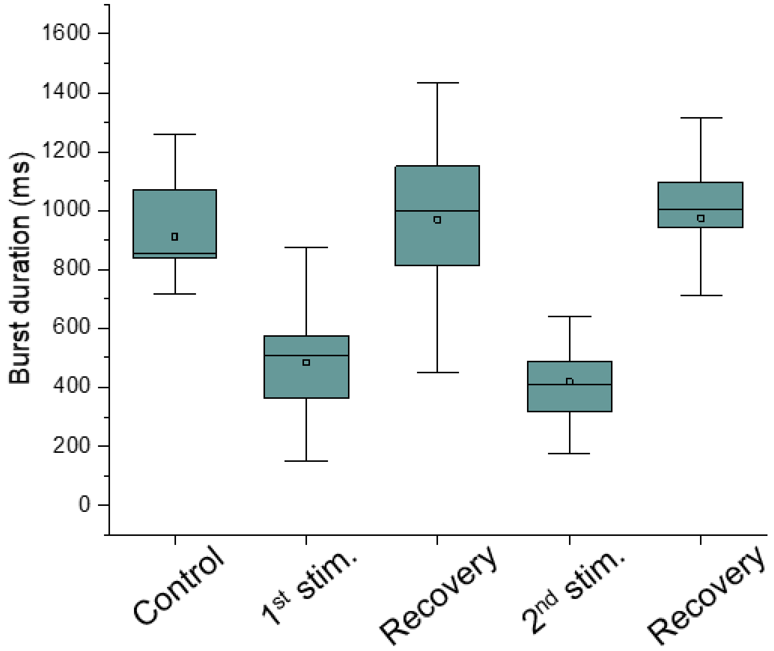


**Figure S9**. Duration of neuronal bursts in each phase of the stimulation experiment: basal, 1st stimulation, 1st recovery of spontaneous bursting, 2nd stimulation, and 2nd recovery of spontaneous bursting.

## 10. Instantaneous frames of spontaneous activity and stimulated spiral wave-front

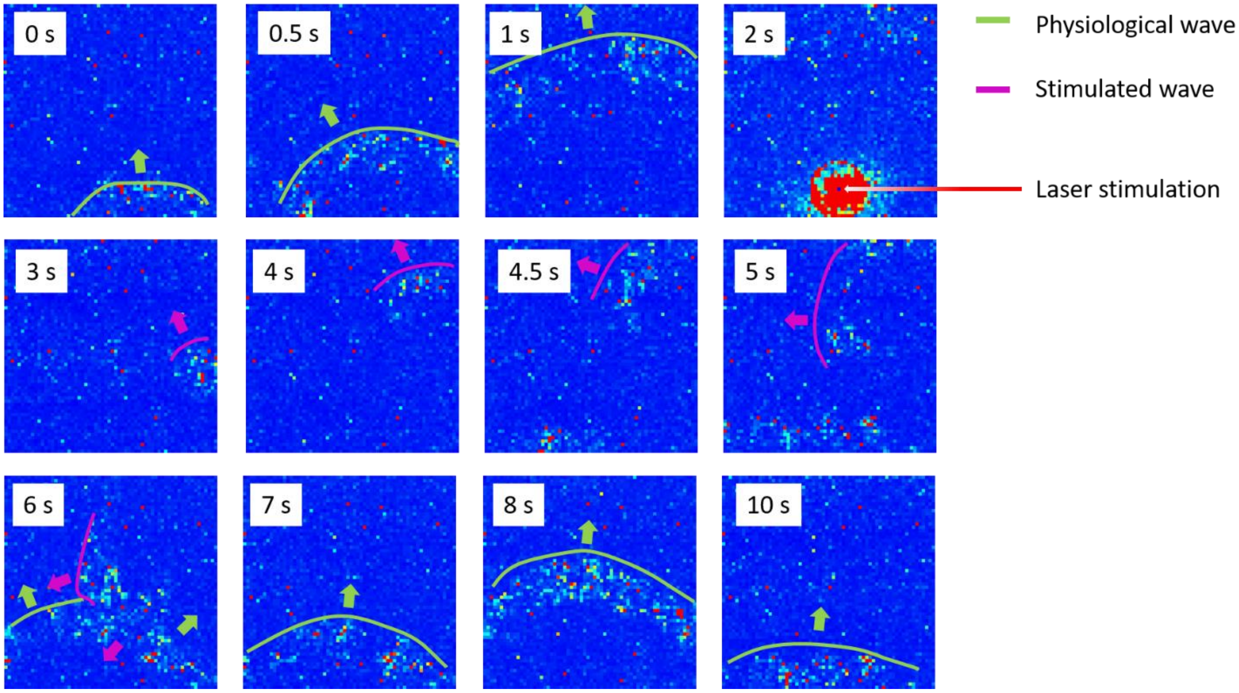


**Figure S10**. Instantaneous frames of spontaneous (highlighted in green) and spiral stimulated activity (pink) propagation in HL-1. The arrow indicates the propagation wave direction. The screenshots and the timeline refer to Movie 05 which can be found as supporting material.

11. Long-term photocurrent measurements with multiple events

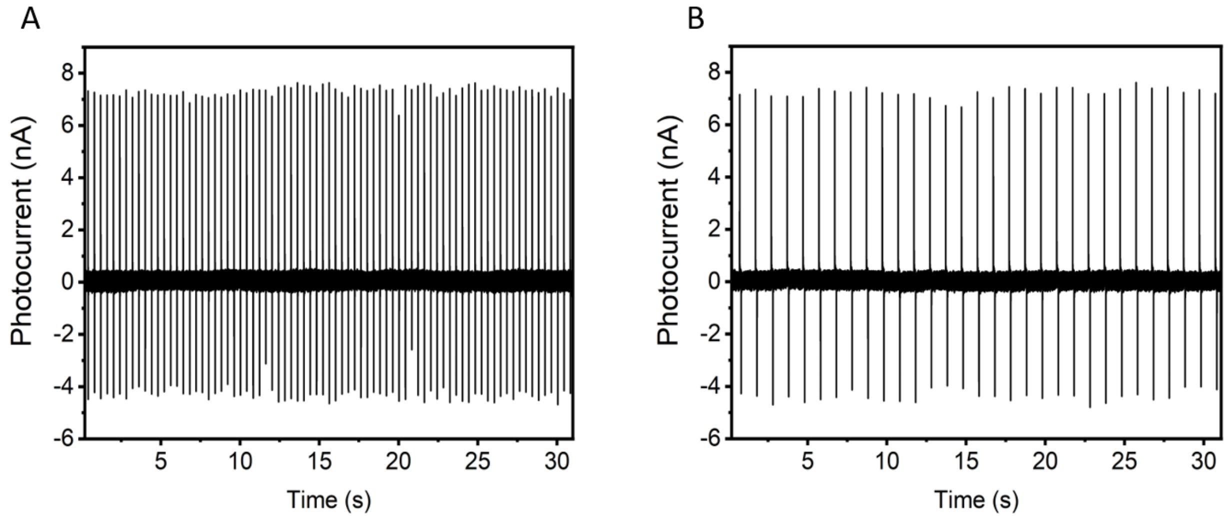


**Figure S11**. 30 seconds long recordings of photocurrent events. A) 10 ms and B) 100 ms laser pulses at 9 mW laser intensity on the MEA electrode surface.
